# Association between atherogenic index of plasma and depression in patients with type 2 diabetes mellitus: A cross-sectional study

**DOI:** 10.1097/MD.0000000000050889

**Published:** 2026-09-25

**Authors:** Xuyang Feng, Lei Ding, Xi Wu

**Affiliations:** a The Second Clinical Medical College, Beijing University of Chinese Medicine, Beijing, China; b Dongfang Hospital, Beijing University of Chinese Medicine, Beijing, China.

**Keywords:** atherogenic index of plasma, cross-sectional study, depressive symptoms, NHANES, type 2 diabetes mellitus

## Abstract

Depression is a major global public health issue and is particularly prevalent but often underrecognized among patients with type 2 diabetes mellitus (T2DM). Metabolic dysregulation has been closely linked to depressive symptoms, and the atherogenic index of plasma (AIP), a marker of dyslipidemia, may be associated with depressive symptoms. However, evidence regarding this association in T2DM populations remains limited. This study aimed to investigate the association between AIP and the risk of depressive symptoms in patients with T2DM using data from the National Health and Nutrition Examination Survey 2011 to 2016. Data were obtained from National Health and Nutrition Examination Survey 2011 to 2016 and included participants with T2DM. AIP was the primary exposure, and clinically significant depressive symptoms were assessed using the Patient Health Questionnaire-9. Multivariable logistic regression was used to evaluate the association between AIP and depressive symptoms. Smooth curve fitting was applied to examine potential nonlinearity, and receiver operating characteristic curve analysis was performed to assess the discriminative ability of AIP. Subgroup and interaction analyses were conducted to test the robustness of the association. A total of 1439 patients with T2DM were included, of whom 180 (12.51%) had clinically significant depressive symptoms. Higher AIP was associated with approximately 2-fold higher odds of depressive symptoms after adjustment for covariates in the adjust II model (odds ratio = 2.0, 95% confidence interval: 1.1–3.6, *P* = .021). Receiver operating characteristic analysis showed statistically significant but limited discriminative ability of AIP for depressive symptoms, with an area under the curve of 0.5781 (95% confidence interval: 0.5341–0.6221). Subgroup analyses showed consistent associations across sex, age, education level, smoking status, and lipid levels, with no significant interactions (*P* for interaction >.05). Smooth curve fitting showed no evidence of nonlinearity, suggesting a linear positive association between AIP and depressive symptoms (*P* for nonlinearity = .213). In patients with T2DM, higher AIP levels were significantly associated with increased risk of depressive symptoms. AIP may serve as a supplementary indicator for assessing the risk of depressive symptoms in this population.

**Key points**

Atherogenic index of plasma is significantly positively associated with the risk of depressive symptoms in patients with type 2 diabetes mellitus.

Atherogenic index of plasma may identify the risk of depressive symptoms not detected by traditional lipid markers.

Atherogenic index of plasma remains associated with depressive symptoms even among individuals with normal traditional lipid profiles.

## 1. Introduction

Depression is a common psychiatric disorder characterized by persistent low mood, loss of interest, cognitive dysfunction, and a variety of emotional and somatic symptoms.^[1]^ With the acceleration of modern lifestyles and increasing social stress, the prevalence of depression continues to rise. Over 300 million people are affected by depression globally each year, making it one of the most significant public health issues contributing to the global disease burden.^[2,3]^ The pathogenesis of depression is complex, involving multiple genetic, psychological, social, and biological factors, including abnormal inflammatory responses, dysregulation of the hypothalamic–pituitary–adrenal (HPA) axis, and changes in metabolic status.^[4–7]^

Studies have shown that patients with type 2 diabetes mellitus (T2DM) are at significantly higher risk of developing depression compared to the general population. This increased risk may be related to the physiological burden of chronic illness, long-term pharmacological treatment, lifestyle restrictions, and neuroendocrine disturbances caused by fluctuations in blood glucose levels.^[8,9]^ Depression not only severely impacts the quality of life in patients with T2DM but also reduces adherence to diabetes management, further exacerbating disease progression and creating a vicious cycle. Therefore, addressing the mental health of patients with T2DM and identifying novel modifiable risk factors for early recognition and prevention of depressive symptoms is of great clinical importance.

In recent years, an increasing body of evidence has demonstrated a close link between metabolic dysregulation and depression, with lipid metabolism disorders potentially playing a key role in its pathophysiology.^[10,11]^ Several National Health and Nutrition Examination Survey (NHANES)-based analyses have also examined the associations between dietary or metabolic indices and depressive symptoms in U.S. adults, further highlighting the metabolic underpinnings of depressive symptoms.^[12]^ The atherogenic index of plasma (AIP), a composite lipid index calculated from the ratio of triglycerides (TG) to high-density lipoprotein cholesterol (HDL-C), is widely used to assess cardiovascular and metabolic risks.^[13,14]^ Previous studies have shown that AIP is not only associated with atherosclerosis, metabolic syndrome, and T2DM, but may also reflect the role of inflammatory responses and oxidative stress mechanisms in the pathogenesis of depression. Although recent studies have reported a positive association between AIP and depressive symptoms among adults with diabetes, most available evidence has focused on diabetes as a broad category. Further evidence specifically focusing on patients with T2DM and evaluating whether AIP provides supplementary information beyond traditional lipid markers remains warranted.

Therefore, this study aims to systematically assess the association between AIP and clinically significant depressive symptoms in T2DM patients based on large sample data from the NHANES database. We hypothesized that higher AIP levels would be independently associated with greater odds of clinically significant depressive symptoms among patients with T2DM. Clarifying this association may provide new insights and scientific evidence for the early screening and intervention of depressive symptoms in this high-risk population.

## 2. Materials and methods

### 2.1. Data source

Data for the present analysis were obtained from the NHANES, which is administered by the National Center for Health Statistics (NCHS). NHANES applies a complex, multistage probability sampling framework to ensure that the noninstitutionalized U.S. population is well represented. Information is collected through interviews, physical assessments, and laboratory measurements. Ethical approval for NHANES was granted by the NCHS Research Ethics Review Board, and all participants provided written informed consent at the time of enrollment. Because the present study was a secondary analysis of publicly available, de-identified NHANES data, no additional ethical approval was required. NHANES data and study protocols are publicly available at https://www.cdc.gov/nchs/nhanes/. The reporting of this study adheres to the Strengthening the Reporting of Observational Studies in Epidemiology guidelines for cross-sectional studies.

### 2.2. Study population

From the 2011 to 2016 NHANES cycles, a total of 29,902 individuals were initially screened. Participants were excluded if they were under 20 years of age, reported pregnancy, did not meet criteria for T2DM, lacked TG values for AIP calculation, had missing Patient Health Questionnaire-9 (PHQ-9) data, or had incomplete demographic information. After these exclusions, 1439 eligible adults with T2DM remained for the final analysis (Fig. 1). Diabetes mellitus was defined if any of the following criteria were met: glycated hemoglobin A1c (HbA1c) ≥6.5%; 2-hour plasma glucose ≥200 mg/dL during an oral glucose tolerance test; fasting plasma glucose ≥126 mg/dL; self-reported physician diagnosis of diabetes; or self-reported use of insulin or other diabetes medications. To minimize the inclusion of participants with T1DM, participants were excluded as probable T1DM if they reported a diagnosis of diabetes before 30 years of age and reported current insulin use without the use of oral hypoglycemic medications. The remaining adult participants with diabetes were considered to have T2DM for the present analysis.

**Figure 1.**
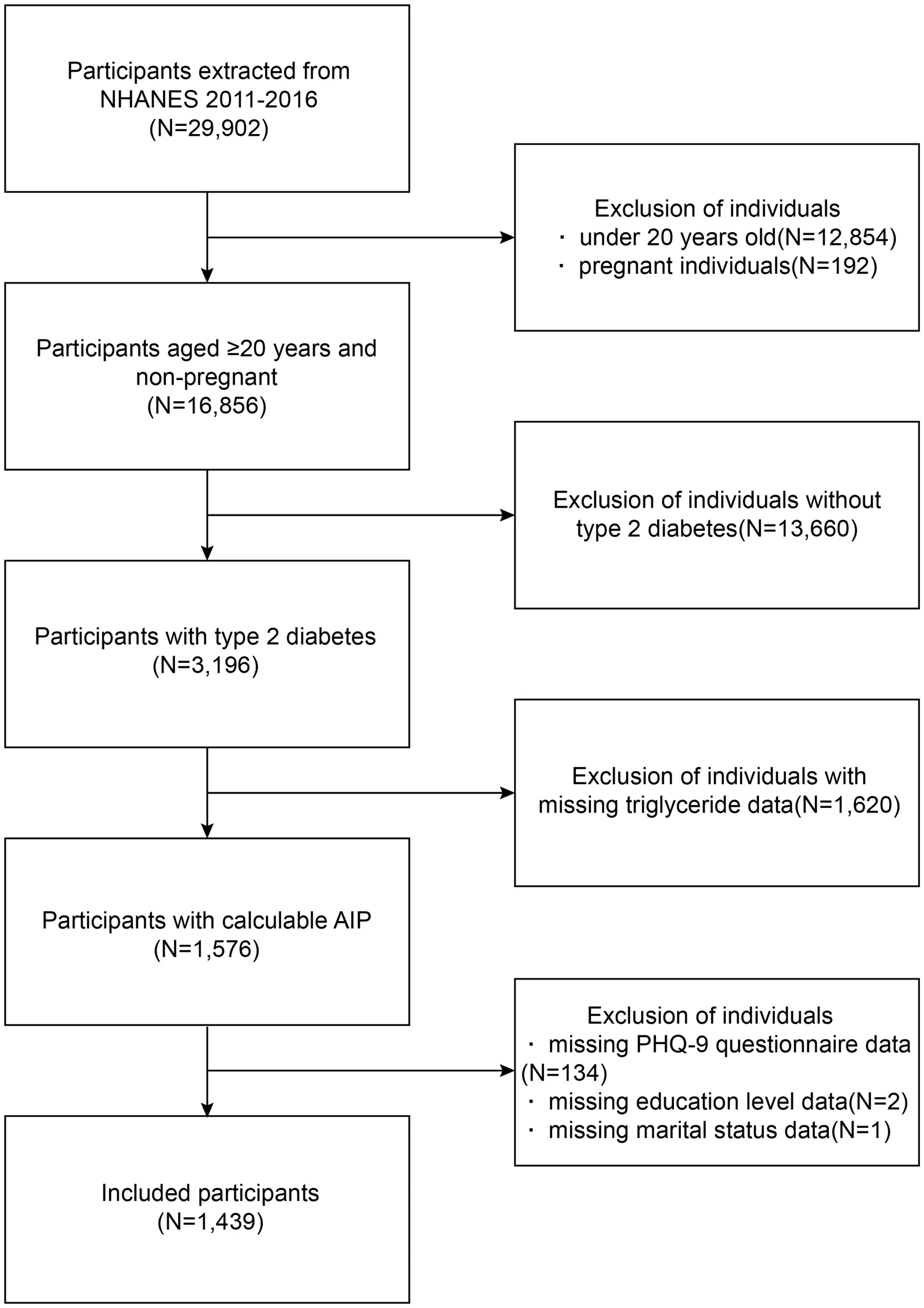
Flowchart of NHANES (2011–2016) participant selection. NHANES = National Health and Nutrition Examination Survey, PHQ-9 = Patient Health Questionnaire-9.

### 2.3. Assessment of AIP

The AIP, the main exposure variable in this study, is a composite lipid indicator calculated from TG and HDL-C levels, reflecting the atherogenic risk in plasma. The formula is:


$${\text{A}IP=log}_{\text{10}}(\text{T}G/HDL\text{-}C)$$

Both TG and HDL-C were measured in mmol/L. In general, AIP has been used as an indicator of atherogenic dyslipidemia and cardiometabolic risk. According to commonly used reference ranges, AIP values <0.11 are generally considered to indicate low risk, values between 0.11 and 0.21 indicate intermediate risk, and values >0.21 indicate increased risk.^[15]^ Higher AIP values reflect higher TG levels, lower HDL-C levels, or both, suggesting a more atherogenic lipid profile. However, these thresholds may vary across different populations and clinical contexts; therefore, in the present study, AIP was analyzed as a continuous variable and was also categorized into quartiles for descriptive analysis: *Q*1 (≤−0.18), *Q*2 (−0.18 to ≤0.04), *Q*3 (0.04–≤0.24), and *Q*4 (>0.24).

### 2.4. Assessment of depressive symptoms

The outcome variable of this study was the presence of clinically significant depressive symptoms. Depressive symptoms were measured using the PHQ-9, which is based on the Diagnostic and Statistical Manual of Mental Disorders, 5th edition, criteria for major depressive disorder and has been extensively validated in population surveys. Each item is scored on a scale from 0 (not at all) to 3 (nearly every day), giving a total possible score between 0 and 27, with higher values reflecting greater symptom burden. Consistent with established practice, a cutoff score of 10 or above was used to indicate the presence of clinically relevant depressive symptoms.

It should be noted that the PHQ-9 is a symptom-based screening instrument rather than a structured clinical diagnostic interview. Therefore, PHQ-9 scores may be influenced by ongoing treatment, including antidepressant use, which could reduce or modify reported depressive symptoms. Given that antidepressant use may also affect lipid metabolism, this issue was considered in the interpretation of the findings and further addressed in the limitations section.

### 2.5. Covariates

Covariates included sex (male and female), age (≥20 years), race/ethnicity (non-Hispanic White, non-Hispanic Black, Mexican American, and other races including multiracial and other Hispanics), education level (less than high school, high school or equivalent, and above high school), marital status (coupled and single or separated), poverty-income ratio (PIR), body mass index (BMI), waist circumference, total cholesterol, low-density lipoprotein cholesterol (LDL-C), albumin, creatinine, alcohol consumption (average drinks/d in the past 12 months), smoking status (every day, some days, and not at all), sleep duration, antidepressant use, statin use, HbA1c, and antihypertensive use.

### 2.6. Statistical analysis

Continuous variables were expressed as means ± standard deviations, and categorical variables were presented as frequencies and percentages. Differences in baseline characteristics across AIP quartiles were compared using 1-way analysis of variance for continuous variables and the chi-square test for categorical variables. To evaluate the association between the AIP and depressive symptoms, multivariable logistic regression models were applied, and the results were reported as odds ratios (ORs) with 95% confidence intervals (CIs). Three models were constructed: non-adjusted model was unadjusted; adjust I model was adjusted for age, sex, and race; and adjust II model was further adjusted for education level, marital status, PIR, BMI, serum albumin, waist circumference, serum creatinine, LDL-C, sleep duration, total cholesterol, alcohol consumption, smoking status, statin use, HbA1c, and antihypertensive use. A generalized additive model was used to fit smooth curves and explore potential nonlinear relationships between AIP and depressive symptoms. Receiver operating characteristic (ROC) curve analysis was performed to assess the discriminative ability of AIP for depressive symptoms. The area under the curve (AUC) with 95% CI was calculated, and the optimal cutoff value was determined using the Youden index, with corresponding sensitivity and specificity reported. In addition, subgroup analyses were conducted by sex (male/female), age (<60 or ≥60 years), race, marital status, education level, PIR (<1 or ≥1), alcohol consumption (<2 or ≥2 drinks/d), BMI (<25 or ≥25 kg/m^2^), sleep duration (<7, 7–9, or ≥9 hours), total cholesterol (<200, 200–240, or ≥240 mg/dL), and LDL-C (<3.4 or ≥3.4 mmol/L). Interaction terms were introduced to evaluate potential effect modifications. Missing data were handled using multiple imputation in IBM SPSS version 26.0 (IBM Corporation), and the most stable imputed dataset was selected based on reliability testing for subsequent analyses. All statistical analyses were conducted using GraphPad Prism 10 (GraphPad Software, LLC), R software (version 3.4.3; R Foundation for Statistical Computing, http://www.R-project.org) and EmpowerStats (X&Y Solutions, Inc.). A 2-sided *P* value <.05 was considered statistically significant.

## 3. Results

### 3.1. Baseline characteristics of the study population

A total of 1439 participants with T2DM were included in this study. The mean age was 61.1 ± 13.5 years, and 749 (52.1%) were male. The mean AIP value was 0.05 ± 0.34, and 180 participants (12.51%) were identified as having clinically significant depressive symptoms. Participants were categorized into quartiles based on AIP levels: *Q*1 (≤−0.18), *Q*2 (−0.18 to ≤0.04), *Q*3 (0.04–≤0.24), and *Q*4 (>0.24). No significant differences were observed among the quartiles in terms of education level, marital status, PIR, albumin, creatinine, alcohol consumption, or sleep duration (all *P* >* *.05). Compared with *Q*1, the *Q*4 group had a higher proportion of males (45.7% vs 61.0%) and non-Hispanic Whites (26.6% vs 44.4%), but a lower proportion of non-Hispanic Blacks (44.2% vs 12.2%) (all *P* < .001). Additionally, BMI (30.5 ± 8.4–33.2 ± 6.8 kg/m^2^), waist circumference (103.5 ± 17.6–112.5 ± 15.1 cm), total cholesterol (177.1 ± 41.3–201.8 ± 52.7 mg/dL), LDL-C (2.5 ± 0.9–2.9 ± 1.0 mmol/L), and HbA1c (6.7% ± 1.6%–7.5% ± 1.9%) increased significantly with rising AIP levels (all *P* < .001). The proportion of daily smokers was highest in *Q*4 (36.9%, *P* = .015), and the prevalence of depressive symptoms was also significantly higher across increasing AIP quartiles, from 8.7% in *Q*1 to 17.3% in *Q*4 (*P* = .004). In addition, antidepressant use increased from 8.7% in *Q*1 to 18.2% in *Q*4 (*P* = .002), and statin use was also significantly different across AIP quartiles, with the highest proportion observed in *Q*4 (65.0%, *P* < .001). In addition, antihypertensive use increased significantly with increasing AIP levels, rising from 55.5% in *Q*1 to 73.7% in *Q*4 (*P* < .001) (Table 1).

**Table 1:** Baseline characteristics of the study population according to atherogenic index of plasma (AIP) quartiles.

Variable	Total (n = 1439)	*Q*1 (≤−0.18)	*Q*2 (−0.18 to ≤0.04)	*Q*3 (0.04–≤0.24)	*Q*4 (>0.24)	*P* value
Age, yr	61.1 (13.5)	63.2 (13.2)	61.5 (13.8)	62.1 (13.4)	57.9 (13.1)	<.001
Sex, %						<.001
Male	749 (52.1)	158 (45.7)	174 (47.2)	192 (54.1)	225 (61.0)	
Female	690 (47.9)	188 (54.3)	195 (52.8)	163 (45.9)	144 (39.0)	
Race, %						<.001
Mexican American	220 (15.3)	33 (9.5)	62 (16.8)	58 (16.3)	67 (18.2)	
Other	346 (24.0)	68 (19.7)	91 (24.7)	94 (26.5)	93 (25.2)	
Non-Hispanic White	516 (35.9)	92 (26.6)	125 (33.9)	135 (38.0)	164 (44.4)	
Non-Hispanic Black	357 (24.8)	153 (44.2)	91 (24.7)	68 (19.2)	45 (12.2)	
Education level, %						.230
Under high school	444 (30.9)	106 (30.6)	109 (29.5)	111 (31.3)	118 (32.0)	
High school or equivalent	337 (23.4)	73 (21.1)	97 (26.3)	94 (26.5)	73 (19.8)	
Above high school	658 (45.7)	167 (48.3)	163 (44.2)	150 (42.3)	178 (48.2)	
Marital status, %						.084
Coupled	868 (60.3)	190 (54.9)	222 (60.2)	227 (63.9)	229 (62.1)	
Single or separated	571 (39.7)	156 (45.1)	147 (39.8)	128 (36.1)	140 (37.9)	
Poverty-income ratio	2.2 (1.5)	2.3 (1.6)	2.2 (1.5)	2.2 (1.5)	2.2 (1.5)	.591
BMI, kg/m^2^	32.1 (7.5)	30.5 (8.4)	31.7 (7.4)	33.0 (7.2)	33.2 (6.8)	<.001
Waist circumference, cm	108.7 (16.6)	103.5 (17.6)	107.7 (16.4)	111.1 (15.6)	112.5 (15.1)	<.001
Total cholesterol, mg/dL	186.1 (46.9)	177.1 (41.3)	181.0 (42.9)	184.0 (45.8)	201.8 (52.7)	<.001
LDL-C, mmol/L	2.8 (1.0)	2.5 (0.9)	2.8 (1.0)	2.9 (1.0)	2.9 (1.0)	<.001
Albumin, g/dL	4.2 (0.3)	4.1 (0.3)	4.2 (0.3)	4.1 (0.3)	4.2 (0.4)	.093
Creatinine, mg/dL	0.9 (0.8)	1.1 (1.2)	1.0 (0.7)	1.0 (0.6)	0.9 (0.5)	.274
HbA1c, %	7.2 (1.8)	6.7 (1.6)	7.2 (1.9)	7.2 (1.8)	7.5 (1.9)	<.001
Alcohol consumption, drinks/d	2.9 (2.0)	2.8 (2.1)	3.0 (2.0)	2.8 (1.9)	3.1 (2.1)	.316
Smoking status, %						.015
Every day	455 (31.6)	94 (27.2)	123 (33.3)	102 (28.7)	136 (36.9)	
Some days	315 (21.9)	89 (25.7)	87 (23.6)	78 (22.0)	61 (16.5)	
Not at all	669 (46.5)	163 (47.1)	159 (43.1)	175 (49.3)	172 (46.6)	
Sleep duration, h	7.2 (1.6)	7.1 (1.7)	7.1 (1.5)	7.4 (1.5)	7.0 (1.7)	.252
Depressive symptoms, %						.004
Yes	180 (12.5)	30 (8.7)	41 (11.1)	45 (12.7)	64 (17.3)	
No	1259 (87.5)	316 (91.3)	328 (88.9)	310 (87.3)	305 (82.7)	
Antidepressant use, %						.002
Yes	191 (13.3)	30 (8.7)	45 (12.2)	49 (13.8)	67 (18.2)	
No	1248 (86.7)	316 (91.3)	324 (87.8)	306 (86.2)	302 (81.8)	
Statin use, %						<.001
Yes	857 (59.6)	173 (50.0)	233 (63.1)	211 (59.4)	240 (65.0)	
No	582 (40.4)	173 (50.0)	136 (36.9)	144 (40.6)	129 (35.0)	
Antihypertensive use, %						<.001
Yes	971 (67.5)	192 (55.5)	245 (66.4)	262 (73.8)	272 (73.7)	
No	468 (32.5)	154 (44.5)	124 (33.6)	93 (26.2)	97 (26.3)	

Continuous variables are presented as mean (SD), and categorical variables are presented as n (%).

BMI = body mass index, HbA1c = glycated hemoglobin A1c, LDL-C = low-density lipoprotein cholesterol, SD = standard deviation.

### 3.2. Association between AIP and depressive symptoms

In the unadjusted model, AIP was significantly positively associated with depressive symptoms (OR = 2.0, 95% CI: 1.3–3.2, *P* = .002). After adjustment for sex, age, and race, this association remained significant (OR = 2.4, 95% CI: 1.5–3.9, *P* < .001). After additional adjustment for education level, marital status, sleep duration, LDL cholesterol, total cholesterol, smoking status, PIR, alcohol consumption, serum albumin, serum creatinine, BMI, waist circumference, statin use, HbA1c, and antihypertensive use, the positive association remained significant (OR = 2.0, 95% CI: 1.1–3.6, *P* = .021). These findings indicate that higher AIP levels were independently associated with approximately 2-fold higher odds of depressive symptoms among patients with T2DM.

When AIP was categorized into quartiles, with *Q*1 as the reference group, the odds of depressive symptoms were significantly higher in *Q*4 after adjustment for the covariates included in the adjust II model (OR = 2.1, 95% CI: 1.2–3.6, *P* = .008), whereas *Q*2 and *Q*3 were not statistically significant. The test for trend showed a significant positive association between AIP quartiles and depressive symptoms across all 3 models (*P* for trend <.001, <.001, and .005, respectively), suggesting that higher AIP quartiles were associated with increased odds of depressive symptoms (Table 2).

**Table 2:** The association between atherogenic index of plasma (AIP) and depressive symptoms in patients with type 2 diabetes mellitus (T2DM).

Exposure	Non-adjusted	Adjust I	Adjust II
AIP index	2.0 (1.3–3.2) .002	2.4 (1.5–3.9)<.001	2.0 (1.1–3.6) .021
AIP index quartile			
*Q*1	Ref	Ref	Ref
*Q*2	1.3 (0.8–2.2) .277	1.3 (0.8–2.2) .292	1.2 (0.7–2.1) .508
*Q*3	1.5 (0.9–2.5) .088	1.6 (1.0–2.7) .056	1.4 (0.8–2.4) .247
*Q*4	2.2 (1.4–3.5)<.001	2.5 (1.5–4.1)<.001	2.1 (1.2–3.6) .008
*P* for trend	<.001	<.001	.005

Non-adjusted model: No covariates were adjusted.

Adjust I model: Adjusted for sex, age, and race.

Adjust II model: Adjusted for sex, age, race, education level, marital status, sleep duration, LDL cholesterol, total cholesterol, smoking status, PIR, alcohol consumption, serum albumin, serum creatinine, BMI, waist circumference, statin use, HbA1c, and antihypertensive use.

Odds ratios (ORs) are presented with 95% confidence intervals (CIs) and corresponding *P* values.

The reference group for quartiles is *Q*1.

AIP = atherogenic index of plasma, BMI = body mass index, HbA1c = glycated hemoglobin A1c, LDL = low-density lipoprotein, PIR = poverty-income ratio.

In addition, the results of smooth curve fitting and threshold effect analysis indicated no clear evidence of a nonlinear relationship between AIP and depressive symptoms after adjustment for the covariates included in the adjust II model, suggesting an approximately linear positive association between the 2 variables (*P* for nonlinearity = .213) (Fig. 2).

**Figure 2.**
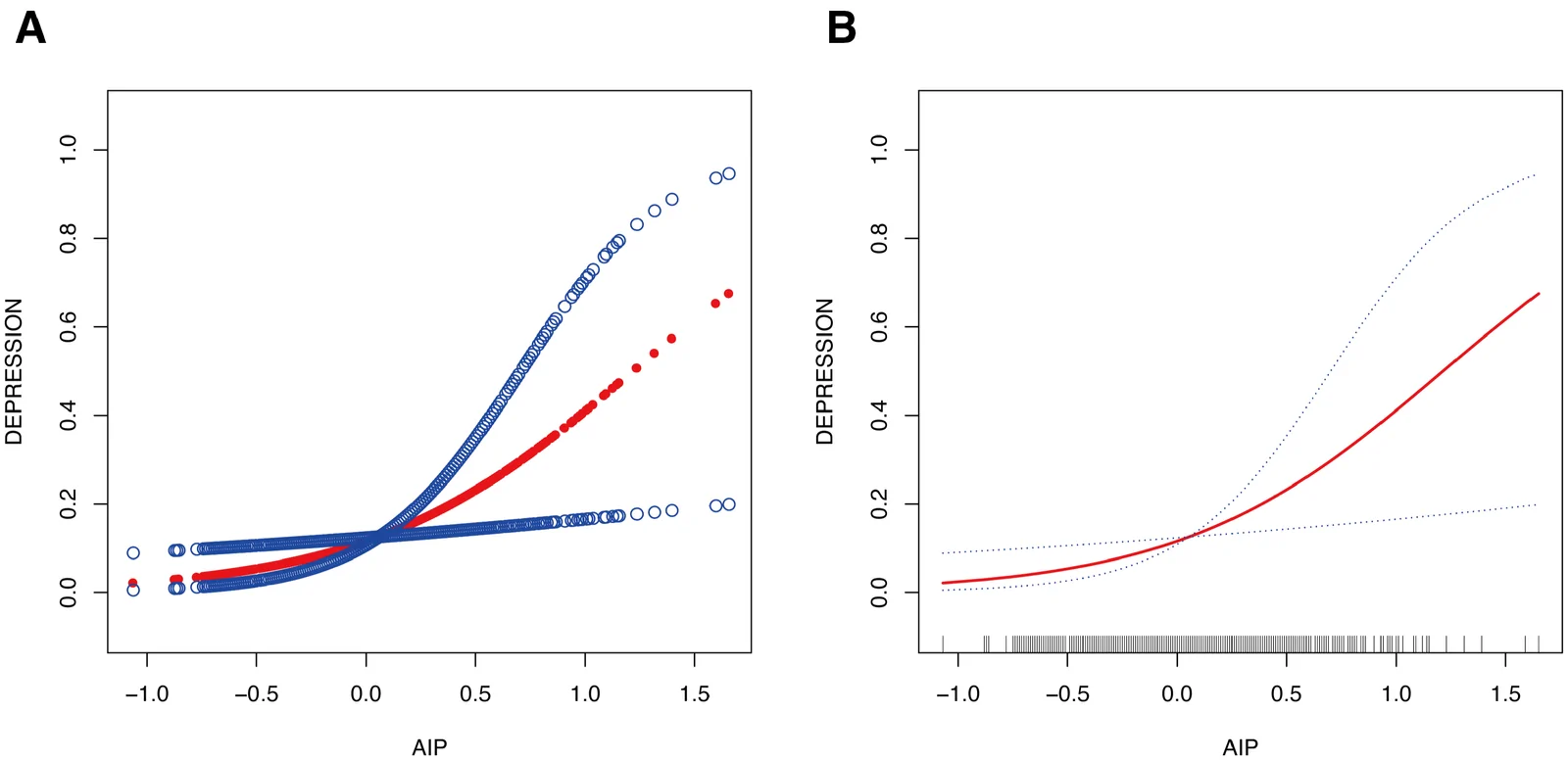
(A) Smooth curve fitting of the association between AIP and depressive symptoms in patients with T2DM. (B) Smooth curve fitting was used to analyze the relationship between AIP and depressive symptoms after adjustment for the covariates included in the adjust II model. The results showed no clear evidence of a complex nonlinear relationship between the 2 variables; instead, an approximately linear positive association was observed. This conclusion was supported by the generalized additive model and threshold effect analysis (*P* for nonlinearity = .213). AIP = atherogenic index of plasma, T2DM = type 2 diabetes mellitus.

### 3.3. ROC curve analysis of AIP for depressive symptoms

ROC curve analysis was further performed to evaluate the discriminative ability of AIP for depressive symptoms in patients with T2DM. The AUC was 0.5781 (95% CI: 0.5341–0.6221, *P* = .0007), indicating that AIP had a statistically significant but limited ability to distinguish participants with depressive symptoms from those without depressive symptoms. The optimal cutoff value based on the Youden index was 0.125, with a sensitivity of 53.33% and a specificity of 61.95% (Fig. 3).

**Figure 3.**
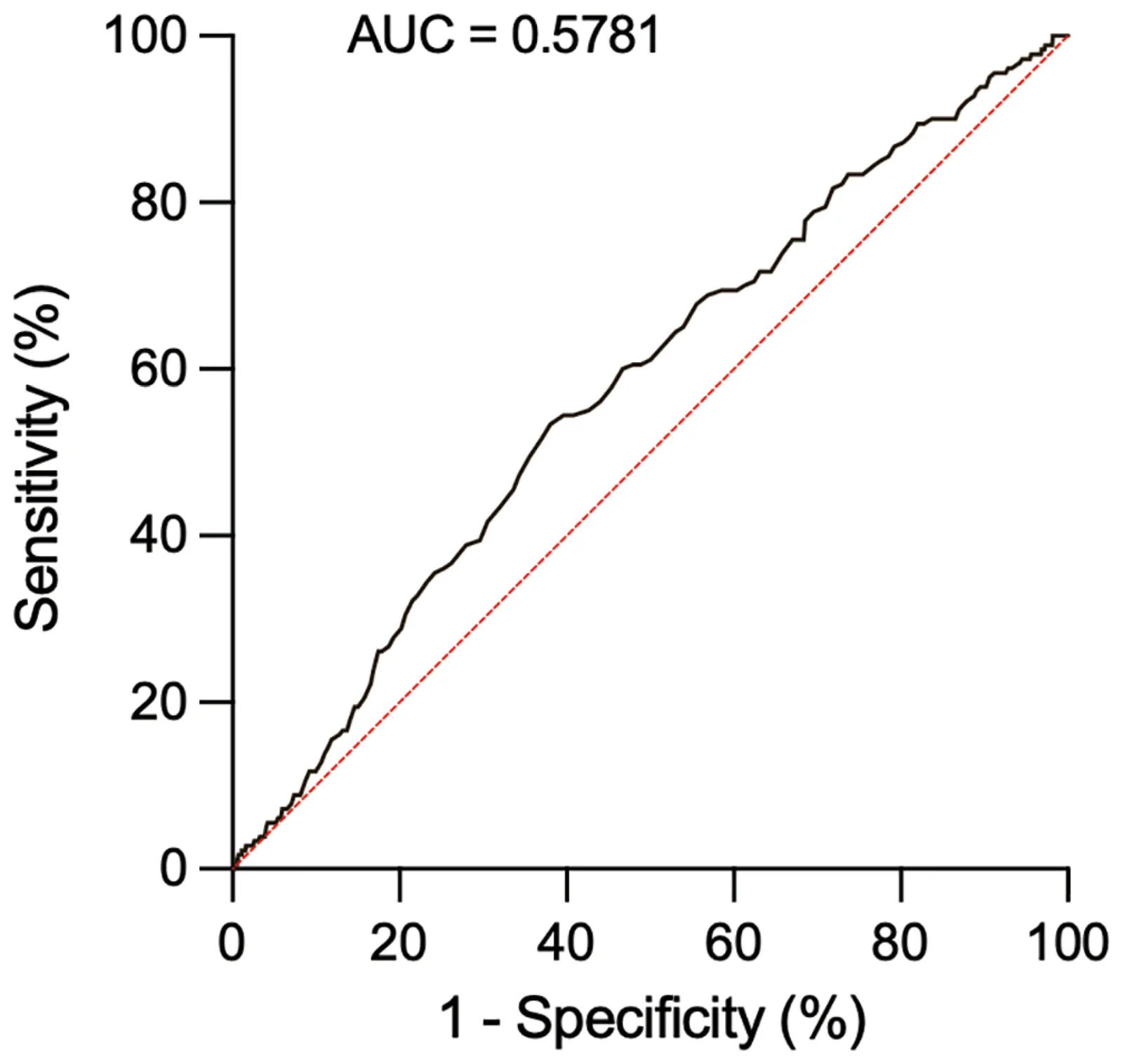
ROC curve of AIP for identifying depressive symptoms in patients with T2DM. ROC curve analysis showed an AUC of 0.5781 (95% CI: 0.5341–0.6221, *P* = .0007). The optimal cutoff value based on the Youden index was 0.125, with a sensitivity of 53.33% and a specificity of 61.95%. AIP = atherogenic index of plasma, AUC = area under the curve, CI = confidence interval, ROC = receiver operating characteristic, T2DM = type 2 diabetes mellitus.

### 3.4. Subgroup analysis

To evaluate the robustness of the association between AIP and depressive symptoms across different subgroups, subgroup analyses were performed. The positive association remained significant in multiple subgroups, particularly among males (OR = 3.6, 95% CI: 1.5–8.5), participants with an education level below high school (OR = 3.1, 95% CI: 1.2–7.8), those with higher socioeconomic status (PIR ≥ 1, OR = 2.3, 95% CI: 1.1–4.7), overweight individuals (BMI ≥ 25, OR = 2.3, 95% CI: 1.3–4.3), daily smokers (OR = 3.3, 95% CI: 1.1–9.7), and those with normal lipid levels (total cholesterol <200 mg/dL, OR = 3.3, 95% CI: 1.7–6.8; LDL-C <3.4mmol/L,OR = 2.4, 95% CI: 1.3–4.7). Moreover, the interaction tests indicated that the *P* for interaction values were all >.05, suggesting no significant effect modification among these subgroups and that the association between AIP and depressive symptoms was relatively stable (Fig. 4).

**Figure 4.**
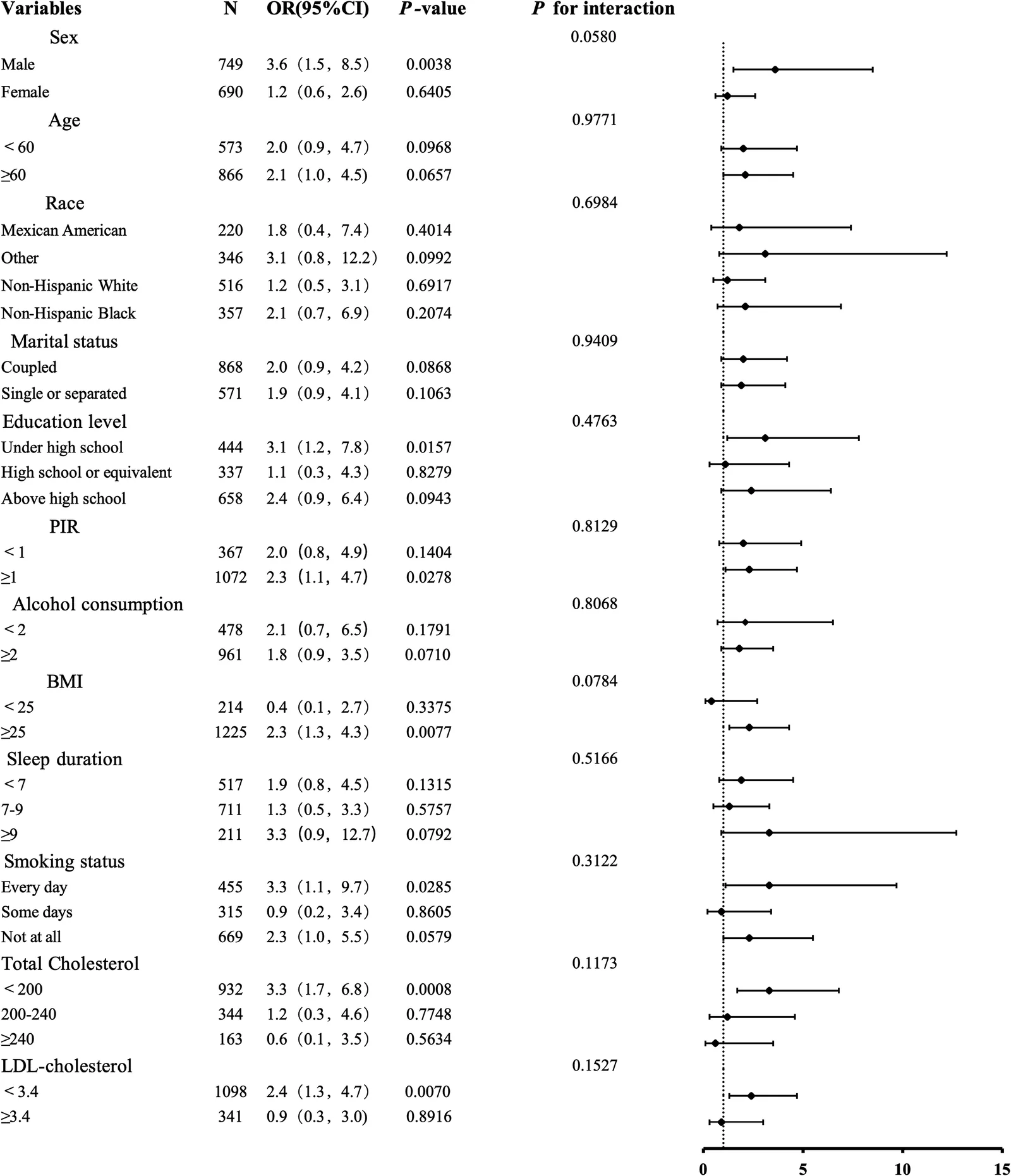
Subgroup analysis of the association between AIP and depressive symptoms in patients with T2DM. Forest plot showing the association between AIP and depressive symptoms across different subgroups stratified by sex, age, race, marital status, education level, PIR, BMI, alcohol consumption, smoking status, sleep duration, and lipid levels, including total cholesterol and LDL-C. No significant interactions were observed, with all *P* values for interaction >.05. AIP = atherogenic index of plasma, BMI = body mass index, CI = confidence interval, LDL-C = low-density lipoprotein cholesterol, OR = odds ratio, PIR = poverty-income ratio, T2DM = type 2 diabetes mellitus.

## 4. Discussion

This study, based on cross-sectional data from 1439 patients with T2DM in the NHANES database, found that higher AIP levels were associated with approximately 2-fold higher odds of depressive symptoms after adjustment for multiple covariates. This association remained stable across subgroups based on sex, education level, PIR, BMI, smoking status, and lipid levels, with no significant interaction effect. In addition, ROC curve analysis showed that AIP had statistically significant but limited discriminative ability for depressive symptoms. These findings suggest that AIP may have potential application value as a supplementary marker for assessing the risk of depressive symptoms in T2DM patients, rather than as a standalone diagnostic tool.

Several previous cross-sectional studies based on NHANES data have reported an association between AIP and depressive symptoms. For instance, Zhang et al^[16]^ identified an L-shaped relationship between AIP and depression based on NHANES 2005 to 2018 data, suggesting the potential of AIP as an indicator for risk assessment of depressive symptoms and that maintaining AIP levels below a certain threshold may help control depressive symptoms. Chen et al^[17]^ found that elevated AIP levels were associated with an increased risk of depressive symptoms in hypertensive patients, indicating that increased atherosclerotic burden may promote the onset of depressive symptoms. Kong et al^[18]^ focused on poststroke patients and found that AIP was a strong metabolic indicator for poststroke depression. Ye et al^[19]^ confirmed a positive correlation between AIP and depressive symptoms in U.S. adults. The results of these studies are consistent with our findings; however, the present study extends previous evidence by focusing specifically on patients with T2DM and by evaluating the supplementary value of AIP beyond traditional lipid markers. Notably, even among individuals with normal lipid profiles, an increase in AIP was still associated with an elevated risk of depressive symptoms, suggesting that AIP may help identify metabolic abnormalities not detected by traditional lipid markers, aiding in the identification of T2DM patients at higher risk for psychological disorders despite the absence of typical metabolic abnormalities. This provides new insights for the early identification of potential high-risk patients.

The onset of depression is the result of multiple interacting factors, and its pathophysiology remains incompletely understood. Existing research primarily focuses on neurotransmitters, neuroendocrine dysfunction, neuroinflammation, and neuroplasticity. Regarding neurotransmitters, serotonin, norepinephrine, and dopamine play key roles in mood regulation. When the function of these neurotransmitters or their receptors is impaired, a decrease in their secretion levels may lead to depressive symptoms.^[20]^ In terms of neuroendocrine dysfunction, depression is also associated with abnormalities in the HPA axis. Abnormal activation of the HPA axis leads to excessive cortisol secretion, disrupting neuroplasticity and affecting the function and structure of brain regions such as the hippocampus, thereby exacerbating depressive symptoms.^[21]^ Additionally, neuroinflammation is considered a critical pathological mechanism in depression. Studies have found that depressed patients often exhibit low-grade systemic inflammation, as evidenced by elevated levels of inflammatory markers such as C-reactive protein and tumor necrosis factor-α. This inflammation activates microglia, impairs neuronal function, reduces the production of neuroprotective factors, and promotes the development of depressive symptoms.^[22,23]^ Alterations in neuroplasticity are also a key mechanism of depression, with patients typically exhibiting reduced brain connectivity, especially in regions related to mood regulation and cognitive function, such as the prefrontal cortex and hippocampus.^[24]^

AIP, as a comprehensive lipid index, is calculated from the ratio of TG to HDL-C, reflecting the overall state of lipid metabolism. Elevated AIP indicates lipid metabolism abnormalities, which are closely related to cardiovascular diseases, metabolic syndrome, and diabetes.^[25–27]^ Furthermore, elevated AIP is often associated with increased systemic inflammation and oxidative stress, which not only damage the cardiovascular system but also affect the function of the central nervous system, thereby contributing to the onset of depressive symptoms.^[28,29]^

The positive correlation between AIP and depressive symptoms may operate through several biological mechanisms. First, elevated AIP reflects lipid abnormalities, particularly increased TG and reduced HDL-C. These changes activate microglia, triggering the release of inflammatory cytokines, enhancing central nervous system inflammation, damaging neurons, and inhibiting neuroplasticity, thus increasing the risk of depressive symptoms.^[30]^ Second, elevated AIP may reflect increased oxidative stress. Elevated TG levels promote the release of free fatty acids, which, when oxidized, produce excessive reactive oxygen species and free radicals that damage cell membranes and neurons. Lower HDL-C levels impair the body’s antioxidant defense capabilities, exacerbating oxidative stress,^[31–33]^ which eventually leads to the onset of depressive symptoms.^[34–36]^ Moreover, studies have shown that elevated TG, reduced HDL-C, and chronic inflammation are associated with HPA axis dysfunction, suggesting that elevated AIP may indirectly reflect HPA axis dysregulation, contributing to the development of depression, although this hypothesis requires further investigation.^[37]^ Finally, AIP may also contribute to depressive symptoms through atherosclerotic-induced vascular damage and impaired cerebral perfusion, which affect brain regions associated with mood regulation, such as the hippocampus and prefrontal cortex, thereby promoting the development of depressive symptoms.^[38–40]^ Moreover, it should be noted that the relationship between AIP and depressive symptoms may be bidirectional. In addition to the potential contribution of dyslipidemia and elevated AIP to depressive symptoms, depression itself and its pharmacological treatment may also influence lipid metabolism. Previous studies have suggested that some antidepressants, including selective serotonin reuptake inhibitors and tricyclic antidepressants, may affect cholesterol and TG metabolism and contribute to changes in lipid profiles.^[41]^ Therefore, elevated AIP levels in some participants may partly reflect the metabolic effects of antidepressant use. Given the cross-sectional design of this study, we could not determine whether depressive symptoms or dyslipidemia occurred 1st, and this issue should be further clarified in future longitudinal studies.

The ROC curve analysis in the present study further suggested that AIP had a statistically significant but limited ability to distinguish T2DM patients with depressive symptoms from those without depressive symptoms. The AUC value of 0.5781 indicates that AIP alone has only modest discriminatory performance. This result is not unexpected because depression is a multifactorial condition influenced by biological, psychological, behavioral, and social factors, and a single lipid-derived index is unlikely to provide strong diagnostic discrimination. Therefore, AIP should not be interpreted as an independent diagnostic marker for depressive symptoms. Instead, it may serve as a supplementary metabolic indicator to help identify T2DM patients who may require closer psychological assessment, especially when considered together with other clinical and psychosocial risk factors.

In summary, this study provides additional evidence supporting a significant positive association between AIP levels and depressive symptoms in patients with T2DM, with generally consistent findings across different subgroups, indicating robust results. Even in patients with normal traditional lipid markers, elevated AIP is still associated with an increased risk of depressive symptoms, suggesting that AIP can detect metabolic abnormalities that are not identified by conventional lipid markers, providing new perspectives for the early identification of potential high-risk individuals in the T2DM population.

Although this study provides valuable findings, several limitations should be acknowledged. First, the cross-sectional design limits the ability to infer causality between AIP and depressive symptoms, and potential temporal ambiguity may exist. In addition, approximately half of the eligible participants with T2DM were excluded because of missing TG data required for AIP calculation, which may have introduced selection bias and affected the representativeness of the study population. Second, although statin use was included as a covariate in the adjusted model because of its direct influence on lipid-related indicators, antidepressant use was only described in the baseline characteristics and was not included in the primary adjusted model. Given that antidepressant treatment may influence both PHQ-9 scores and lipid metabolism, residual confounding related to antidepressant use could not be fully excluded. In addition, detailed information on antidepressant type, dosage, treatment duration, and medication adherence was not available in the NHANES database, which may further limit the interpretation of medication-related effects. Third, several potentially important confounders, including physical activity, C-reactive protein, vitamin D levels, and genetic factors, were not included in the final analyses. Therefore, residual confounding cannot be completely excluded, and missing data on some variables may also limit the comprehensiveness of the analysis and the generalizability of the conclusions. Fourth, some PHQ-9 items overlap with T2DM-related somatic symptoms, such as fatigue and sleep disturbances, which may have led to an overestimation of depressive symptom prevalence in this population. Finally, multiple subgroup analyses were performed without formal correction for multiple comparisons. Therefore, some statistically significant subgroup findings may be attributable to chance and should be interpreted as exploratory and hypothesis-generating rather than confirmatory.

## 5. Conclusion

In patients with T2DM, higher AIP levels were significantly associated with higher odds of depressive symptoms, and this association was generally consistent across different subgroups. AIP may serve as a supplementary marker for the early identification of T2DM patients at elevated risk of depressive symptoms; however, prospective studies are warranted to confirm these findings and elucidate the underlying mechanisms.

## Acknowledgments

The authors would like to thank the National Health and Nutrition Examination Survey (NHANES) team for providing access to the data.

## Author contributions

**Conceptualization:** Xuyang Feng.

**Funding acquisition:** Xi Wu.

**Data curation:** Xuyang Feng.

**Formal analysis:** Xuyang Feng.

**Methodology:** Lei Ding.

**Project administration:** Lei Ding.

**Resources:** Lei Ding.

**Supervision:** Xi Wu.

**Validation:** Xi Wu.

**Visualization:** Lei Ding, Xi Wu.

**Writing – original draft:** Xuyang Feng, Xi Wu.

**Writing – review & editing:** Xuyang Feng.

## References

[1] GBD 2019 Mental Disorders Collaborators. Global, regional, and national burden of 12 mental disorders in 204 countries and territories, 1990–2019: a systematic analysis for the Global Burden of Disease Study 2019. Lancet Psychiatry. 2022;9:137–50. 10.1016/S2215-0366(21)00395-3 PMC8776563 35026139

[2] Stringaris A. What is depression? J Child Psychol Psychiatry. 2017;58:1287–9. 10.1111/jcpp.12844 29148049

[3] Xiang AH Martinez MP Chow T. Depression and anxiety among US children and young adults. JAMA Network Open. 2024;7:e2436906–e2436906. 10.1001/jamanetworkopen.2024.36906 PMC11445688 39352699

[4] Yin Y Ju T Zeng D. “Inflamed” depression: a review of the interactions between depression and inflammation and current anti-inflammatory strategies for depression. Pharmacol Res. 2024;207:107322. 10.1016/j.phrs.2024.107322 39038630

[5] Qiu W Cai X Zheng C Qiu S Ke H Huang Y. Update on the relationship between depression and neuroendocrine metabolism. Front Neurosci. 2021;15:728810. 10.3389/fnins.2021.728810 PMC8438205 34531719

[6] Sălcudean A Bodo CR Popovici RA. Neuroinflammation – a crucial factor in the pathophysiology of depression – a comprehensive review. Biomolecules. 2025;15:502. 10.3390/biom15040502 PMC12024626 40305200

[7] Lei AA Phang VWX Lee YZ. Chronic stress-associated depressive disorders: the impact of HPA axis dysregulation and neuroinflammation on the hippocampus – a mini review. Int J Mol Sci. 2025;26:2940. 10.3390/ijms26072940 PMC11988747 40243556

[8] Farooqi A Gillies C Sathanapally H. A systematic review and meta-analysis to compare the prevalence of depression between people with and without type 1 and type 2 diabetes. Primary Care Diabetes. 2022;16:1–10. 10.1016/j.pcd.2021.11.001 34810141

[9] Fanelli G Raschi E Hafez G. The interface of depression and diabetes: treatment considerations. Transl Psychiatry. 2025;15:22. 10.1038/s41398-025-03234-5 PMC11760355 39856085

[10] Khalfan AF Campisi SC Lo RF McCrindle BW Korczak DJ. The association between adolescent depression and dyslipidemia. J Affect Disord. 2023;338:239–45. 10.1016/j.jad.2023.06.017 37302507

[11] Chourpiliadis C Zeng Y Lovik A. Metabolic profile and long-term risk of depression, anxiety, and stress-related disorders. JAMA Network Open. 2024;7:e244525. 10.1001/jamanetworkopen.2024.4525 PMC10988352 38564219

[12] Jian X Jian S Zhang Z Ye Y Tang X Huang R. Association between dietary fiber intake and post-stroke depression among US women: insights from the NHANES 2005–2018 cross-sectional study. Front Nutr. 2025;12:1586511. 10.3389/fnut.2025.1586511 PMC12341476 40799518

[13] Andraschko LM Gazi G Leucuta DC Popa S-L Chis BA Ismaiel A. Atherogenic index of plasma in metabolic syndrome – a systematic review and meta-analysis. Medicina (Kaunas, Lithuania). 2025;61:611. 10.3390/medicina61040611 PMC12028871 40282902

[14] Wu TT Gao Y Zheng YY Ma Y-T Xie X. Atherogenic index of plasma (AIP): a novel predictive indicator for the coronary artery disease in postmenopausal women. Lipids Health Dis. 2018;17:197. 10.1186/s12944-018-0828-z PMC6106932 30134981

[15] Kurklu HA Tan TS Ozyuncu N Baskovski E Ozdol C. Atherogenic index of plasma predicts obstructive coronary artery disease in patients with stable angina pectoris. Diagnostics (Basel, Switzerland). 2023;13:3249. 10.3390/diagnostics13203249 PMC10606625 37892070

[16] Zhang H Zhang G Fu J. Exploring the L-shaped relationship between atherogenic index of plasma and depression: results from NHANES 2005–2018. J Affect Disord. 2024;359:133–9. 10.1016/j.jad.2024.05.073 38768824

[17] Chen T Qian Y Deng X. Relationship between atherosclerotic burden and depressive symptoms in hypertensive patients: a cross-sectional study based on the NHANES database. J Affect Disord. 2024;361:612–9. 10.1016/j.jad.2024.06.087 38925305

[18] Kong D Zou W. Association between atherogenic index of plasma and post-stroke depression: a cross-sectional study. Eur J Psychotraumatol. 2024;15:2429266. 10.1080/20008066.2024.2429266 PMC11632925 39648850

[19] Ye Z Huang W Li J Tang Y Shao K Xiong Y. Association between atherogenic index of plasma and depressive symptoms in US adults: results from the National Health and Nutrition Examination Survey 2005 to 2018. J Affect Disord. 2024;356:239–47. 10.1016/j.jad.2024.04.045 38608770

[20] Blier P. Neurotransmitter targeting in the treatment of depression. J Clin Psychiatry. 2013;74(suppl 2):19–24. 10.4088/JCP.12084su1c.04 24191974

[21] Menke A. The HPA axis as target for depression. Curr Neuropharmacol. 2024;22:904–15. 10.2174/1570159X21666230811141557 PMC10845091 37581323

[22] Troubat R Barone P Leman S. Neuroinflammation and depression: a review. Eur J Neurosci. 2021;53:151–71. 10.1111/ejn.14720 32150310

[23] Li Q Xie Y Lin J. Microglia sing the prelude of neuroinflammation-associated depression. Mol Neurobiol. 2025;62:5311–32. 10.1007/s12035-024-04575-w 39535682

[24] Rădulescu I Drăgoi AM Trifu SC Cristea MB. Neuroplasticity and depression: rewiring the brain’s networks through pharmacological therapy. Exp Therap Med. 2021;22:1131. 10.3892/etm.2021.10565 PMC8383338 34504581

[25] Niroumand S Khajedaluee M Khadem-Rezaiyan M. Atherogenic index of plasma (AIP): a marker of cardiovascular disease. Med J Islamic Republic Iran. 2015;29:240. PMC4715400 26793631

[26] Li YW Kao TW Chang PK Chen W-L Wu L-W. Atherogenic index of plasma as predictors for metabolic syndrome, hypertension and diabetes mellitus in Taiwan citizens: a 9-year longitudinal study. Sci Rep. 2021;11:9900. 10.1038/s41598-021-89307-z PMC8110777 33972652

[27] Zhu XW Deng FY Lei SF. Meta-analysis of atherogenic index of plasma and other lipid parameters in relation to risk of type 2 diabetes mellitus. Primary Care Diabetes. 2015;9:60–7. 10.1016/j.pcd.2014.03.007 24810146

[28] Liu Z Zhang L Wang L. The predictive value of cumulative atherogenic index of plasma (AIP) for cardiovascular outcomes: a prospective community-based cohort study. Cardiovasc Diabetol. 2024;23:264. 10.1186/s12933-024-02350-8 PMC11264486 39026310

[29] Tao S Yu L Li J. Higher atherogenic index of plasma is associated with increased major depressive disorder: insights from a nationally representative study. Front Psychiatry. 2024;15:1441119. 10.3389/fpsyt.2024.1441119 PMC11499100 39450305

[30] Ebtehaj S Gruppen EG Parvizi M Tietge UJF Dullaart RPF. The anti-inflammatory function of HDL is impaired in type 2 diabetes: role of hyperglycemia, paraoxonase-1 and low grade inflammation. Cardiovasc Diabetol. 2017;16:132. 10.1186/s12933-017-0613-8 PMC5639738 29025405

[31] Wang L Sapuri-Butti AR Aung HH Parikh AN Rutledge JC. Triglyceride-rich lipoprotein lipolysis increases aggregation of endothelial cell membrane microdomains and produces reactive oxygen species. Am J Physiol Heart Circ Physiol. 2008;295:H237–44. 10.1152/ajpheart.01366.2007 PMC2494756 18487440

[32] Wang L Gill R Pedersen TL Higgins LJ Newman JW Rutledge JC. Triglyceride-rich lipoprotein lipolysis releases neutral and oxidized FFAs that induce endothelial cell inflammation. J Lipid Res. 2009;50:204–13. 10.1194/jlr.M700505-JLR200 PMC2636918 18812596

[33] Feng J Wang Y Li W. High levels of oxidized fatty acids in HDL impair the antioxidant function of HDL in patients with diabetes. Front Endocrinol. 2022;13:993193. 10.3389/fendo.2022.993193 PMC9630736 36339401

[34] Atigh MBG Ghorbani A Zarban A. Comparison between oxidative stress (8-OhdG, MDA) and atherogenic risk (AIP) factors in type 2 diabetics and healthy people: a case-control study. J Transl Med Res. 2017;24:42–9.

[35] Nawrocka-Rutkowska J Szydłowska I Jakubowska K. The role of oxidative stress in the risk of cardiovascular disease and identification of risk factors using AIP and castelli atherogenicity indicators in patients with PCOS. Biomedicines. 2022;10:1700. 10.3390/biomedicines10071700 PMC9312468 35885005

[36] Feng X Zhang W Liu X. Ferroptosis-associated signaling pathways and therapeutic approaches in depression. Front Neurosci. 2025;19:1559597. 10.3389/fnins.2025.1559597 PMC11961976 40177374

[37] Luan X Wang X Shi Y. Abnormalities of lipid metabolism in the progression and treatment of depression. Front Psychiatry. 2025;16:1589663. 10.3389/fpsyt.2025.1589663 PMC12161997 40511456

[38] Zhou J Zhang C Wu X. Identification of genes and pathways related to atherosclerosis comorbidity and depressive behavior via RNA-seq and bioinformation analysis in ApoE−/− mice. Ann Transl Med. 2019;7:733. 10.21037/atm.2019.11.118 PMC6990041 32042749

[39] Peterson BS Li J Trujillo M. A multi-site 99mTc-HMPAO SPECT study of cerebral blood flow in a community sample of patients with major depression. Transl Psychiatry. 2024;14:234. 10.1038/s41398-024-02961-5 PMC11148018 38830866

[40] Chrysohoou C Kollia N Tousoulis D. The link between depression and atherosclerosis through the pathways of inflammation and endothelium dysfunction. Maturitas. 2018;109:1–5. 10.1016/j.maturitas.2017.12.001 29452775

[41] Richards-Belle A Austin-Zimmerman I Wang B. Associations of antidepressants and antipsychotics with lipid parameters: do CYP2C19/CYP2D6 genes play a role? A UK population-based study. J Psychopharmacol (Oxford, England). 2023;37:396–407. 10.1177/02698811231152748 PMC10101178 36772859

